# Estimating the Frequency of an Elongated Styloid Process of the Temporal Bone Along With its Anatomical Variations in Length Using Three-Dimensional Computerized Tomography

**DOI:** 10.7759/cureus.109227

**Published:** 2026-05-19

**Authors:** Sharmistha Sagar, Kumar Satish Ravi, Bijendra K Meena, Amit Modwal

**Affiliations:** 1 Department of Anatomy, National Institute of Medical Sciences and Research, Jaipur, IND; 2 Department of Radiation and Imaging Technology, National Institute of Medical Sciences and Research, Jaipur, IND; 3 Department of Otorhinolaryngology, National Institute of Medical Sciences and Research, Jaipur, IND

**Keywords:** 3d ct, anatomical variation, elongated styloid process, styloid process, temporal bone

## Abstract

Background: The styloid process is a long, slender bone, part of the temporal bone, projecting downward, medially to variable length and angulation on both sides in the same individual and between two individuals, of different sexes and different ages. The object was to estimate the frequency of the elongated styloid process (ESP) of the temporal bone and variations in the length of the styloid process by using three-dimensional computerised tomography (3D CT) on lateral and posterior views in a population ranging from 18 to 80 years.

Material and methods: This is an observational study, conducted in the Department of Anatomy and Radiology of the National Institute of Medical Sciences and Research (NIMS), Jaipur, Rajasthan, India, on 150 3D CT scans showing the styloid process in the posterior and lateral view. The CT scans of the face and paranasal sinus in axial and coronal sections were collected, and a 360-degree 3D image of the face and skull showing the styloid process in lateral and posterior were generated by using the RadiAnt DICOM (Medixant, Poznan, Poland) viewer (64-bit) software. The length of the styloid process was measured with the help of this software. The broken styloid processes were excluded from the study.

Results: Out of 150 3D CT, 113 were male, and 37 were female, age wise 29.3% of the 18 to 40 years age group, 34% of the 41 to 60 years age group, and 36.7% of the 61 to 80 years age group. The frequency of 2.01-3 cm long styloid processes was maximum on both views. The frequency of 3.01 cm to 4.00 cm long and less than 1 cm long styloid processes was significantly different on the lateral and posterior view, but in the rest of all groups, this difference in frequency was not statistically significant.

The mean length of the styloid process on the right posterior view was 2.929 cm, SD=0.829; on the left posterior view, 2.965 cm, SD=0.904; on the right lateral view, 2.520 cm, SD=0.851; on the left lateral view, 2.569 cm, SD=0.869. The overall frequency of the ESP on the lateral view was 30%; in contrast, it was 53.3% on the posterior view. This difference in frequency was statistically significant (p value=.000042). In both genders, in all age groups, on both sides, the difference in frequency is not statistically significant on both views. The styloid process was bilaterally elongated on the lateral view in 17.3% of 3D CT and on the posterior view in 34%, and unilaterally elongated 12.7% on the lateral view and 19.35% on the posterior view. This difference is statistically significant with P-value=.004075.

Conclusion: The length of the posterior view of 3D CT should be used to estimate the frequency of ESP. The elongation of the styloid more than 3 cm is not only a criterion for the occurrence of symptoms of Eagle’s syndrome, but also the angulation. The frequency of occurrence of symptoms increases with size, more than 4 cm. This also explains the lesser prevalence of development of symptoms of Eagle’s syndrome.

## Introduction

The styloid process is a cylindrical, needle-like projection of variable length. It is part of the petrous temporal bone and is directed downward, medially, and anteriorly. The length of the styloid process varies widely, from very small to elongated, to reach the hyoid bone. This length may vary within the same individual between sides, across genders, and across age groups. It originates from the second pharyngeal arch. It serves as an attachment point for three muscles and two ligaments that together form the stylohyoid apparatus. The muscles of the stylohyoid apparatus are Stylohyoid, Stylopharyngeus, and Styloglossus, and the ligaments are the Stylo-mandibular and the Stylohyoid ligament [1-3]. Significant vessels and nerves surround the styloid process. The internal jugular vein, internal carotid artery, glossopharyngeal nerve (CN IX), vagus nerve (CN X), and accessory nerve (CN XI) lie medial to the styloid process, while hypoglossal and facial nerves lie lateral to it. Long-term unilateral or bilateral pharyngeal, neck, or throat pain; a foreign body sensation in the throat; odynophagia; dysphagia; headache; and ringing or buzzing in the ears can be caused by an elongated styloid process. The more severe form is the vascular form, in which close contact of the styloid process with the carotid artery can cause vascular compression or even tearing, which may lead to stylo-carotid syndrome, stroke, or transient ischemic attack [4-8]. The styloid process can be single, double, or triple [9]. It has been reported to be 8 cm long [10-12]. Various radiological methods are used for morphometric measurement of the styloid process, such as orthopantomography (OPG), cone-beam computed tomography (CBCT), and CT scan, but 3D CT is the gold standard [13-15]. The elongated styloid process (ESP) can show different patterns of calcification [16-18]. Eagle syndrome can be diagnosed using CT, but CT angiography also has a role in aiding diagnosis [19].

The length of the styloid process varies among individuals, ages, and between genders. The present study was conducted on the posterior and lateral views of 150 three-dimensional computed tomography scans to estimate anatomical variations in the length of the styloid process and the frequency of ESP.

## Materials and methods

**Figure 1:**
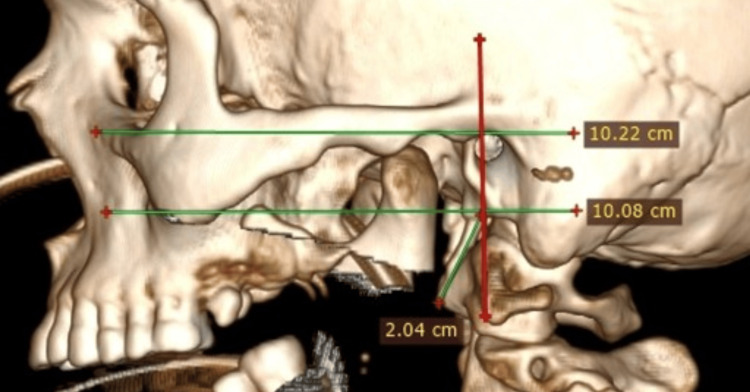
A 3D CT lateral-view image showing the styloid process, a Frankfurt line (upper line), a horizontal line (lower line) drawn at the base of the styloid process, and a vertical line crossing the horizontal line at the centre of the base of the styloid process. 3D CT, three-dimensional computed tomography.

This is an observational study conducted in the departments of anatomy and radiology at the National Institute of Medical Sciences and Research (NIMS), Jaipur, Rajasthan, on 150 3D CT scans of individuals aged 18 to 80 years that show the styloid process in posterior and lateral views. The CT scans of the face and paranasal sinus in axial and coronal sections were collected from the Department of Radiology, and a 360-degree 3D image of the skull was generated by using the RadiAnt DICOM (Medixant, Poznan, Poland) viewer 64-bit software. On a lateral view of the 3D CT image, a superior horizontal line (Frankfurt line) is drawn from the infraorbital margin to the superior-meatal margin of the external auditory canal, and another horizontal line is drawn parallel to it at the base of the styloid process. A vertical line is drawn intersecting a horizontal line at the centre of the base of the styloid process, forming a 90-degree angle with the horizontal line (Figure 1).

**Figure 2:**
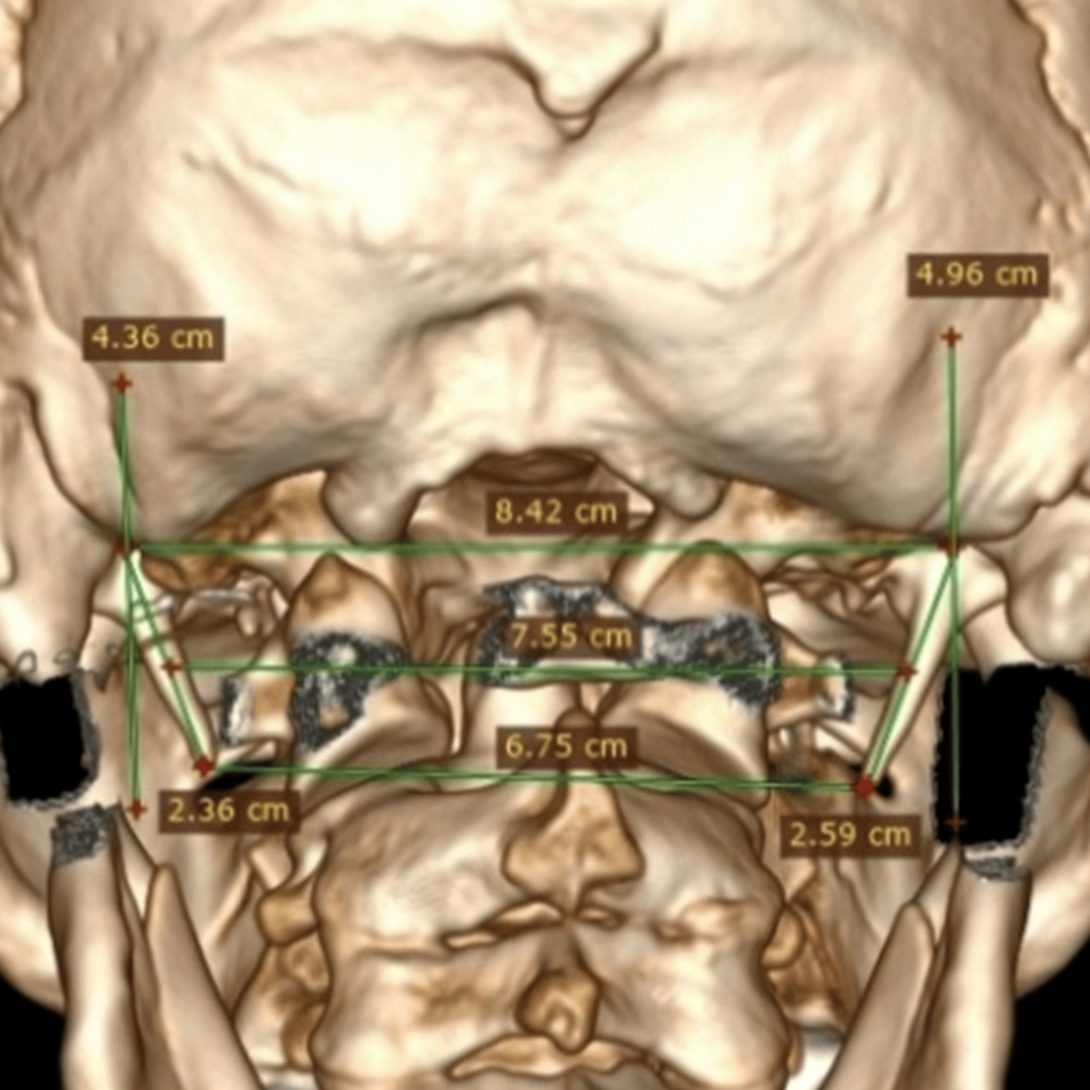
A 3D CT posterior-view image showing a horizontal line drawn at the base of the styloid process and a vertical line passing through the centre of the base of the styloid process. 3D CT, three-dimensional computed tomography.

On the posterior view 3D CT image, a horizontal line is drawn from the centre of the base of each styloid process, where it originates from the temporal bone, and a vertical line is drawn through the centre of the base of the styloid process, making a 90-degree angle with the horizontal line (Figure 2). The length of the styloid process was measured from the centre of the base of the styloid process up to the tip on the lateral and posterior views. The broken styloid processes were excluded from the study. 

For study purposes, the 3D CTs were stratified by gender and age to assess variations in styloid process length and ESP frequency across genders and ages. The different length ranges were measured with a 1 cm gap. The length of the SP up to 3 cm is considered normal length; more than 3 cm is an elongated styloid process. The frequency of ESP was estimated separately for males and females. The frequency of the elongated styloid process was estimated separately for both sides. The frequency of laterality of the elongated styloid process was also estimated. All data was collected in both the lateral and posterior views. Mean length and SD were calculated by using Microsoft Excel (Microsoft Corporation, Redmond, WA, USA) and IBM SPSS Statistics, Version 24 (IBM Corp., Armonk, NY, USA).

Statistical methods: For the analysis, the data obtained were entered into Microsoft Excel (Microsoft Corporation, Redmond, WA, USA). The results and correlations between age group, gender, side, laterality of elongation, and size (cm) were analyzed using IBM SPSS Statistics, Version 24 (IBM Corp., Armonk, NY, USA). In this study, we applied the Chi-square test to assess significant differences, and the p-value was set at <0.05 as the level of significance.

## Results

The 150 CT scans of face and head, and paranasal sinuses, comprising 113 of males (75.4%) and 37 of females (24.6%), aged 18 to 80 years, were collected, and 360 3D CT images were generated by using the RadiAnt DICOM viewer (64-bit) software. The morphometric measurement of the styloid process was done via the RadiAnt DICOM software on the posterior and lateral views of 3D CT. The mean age was 52.32 years (SD = 18.18). Age-wise, 42 (29.3%) 3D CT of 18 to 40 years of age, 51 (34%) were 41 to 60 years of age, and 57 (36.7%) were 61 to 80 years of age (Table 1).

**Table 1:** Shows gender wise and age wise distribution of cases.

Gender	Number	Percentage
Male	113	75.4
female	37	24.6
Age groups		
18 yrs to 40 yrs	42	29.3
41yrs to 60 yrs	51	34
61 yrs to 80 years	57	36.7
Total	150	100

The lengths of 300 styloid processes were measured using software, and data were collected. The frequency of the length of the styloid process was measured at 1 cm intervals. The frequency of 2.01 cm to 3.00 cm long styloid processes was maximum on both views and in elongated and normal styloid processes. The frequency of 3.01 cm to 4.00 cm long styloid processes was maximum in the elongated styloid process (Table 2). On the posterior view 79.68 % and on the lateral view, 73.52 % of the elongated styloid process measured 3.01 to 4.00 cm. The difference in the frequency of the styloid process of less than 3 cm long for the lateral and posterior views is statistically significant (χ² test=7.4603 and P-value = 0.02398 ). The remaining groups were not statistically significant (P-value =0.7825).

**Table 2:** Frequency of different length range of styloid process on lateral and posterior view of 3D CT. 3D CT, three-dimensional computed tomography.

Different ranges of length of styloid process	Lateral view (lateral length)	Posterior view (posterior length)	Chi-square test	P-value	Degree of freedom
Less than 1 cm	13(4.3%)	01 (.3%)	7.4603	0.02398 Significant	2
1 cm to 2.00 cm	42 (14%)	32 (10.7%)			
2.01 cm to 3.00 cm	177 (59%)	139 (46.3%)			
3.01 cm to 4 cm	50 (16.7%)	102 (34%)	1.0775	0.7825	3
4.01 cm to 5 cm	12 (4.00%)	17 (5.7%)			
5.01 cm to 6 cm	05 (1.7%)	08 (2.7%)			
More than 6 cm	01 (.3%)	01 (.3%)			
Total	300 (100%)	300 (100%)			

The mean lengths of the styloid processes on both sides across both genders and age groups were calculated using IBM SPSS Statistics, Version 24 (IBM Corp., Armonk, NY, USA). It was higher in males than in females across all groups, but the difference was not significant (Table 3).

**Table 3:** Mean length of styloid process with standard deviation on posterior and lateral view on both sides, in both genders.

Side and gender	Mean length on lateral view	Mean length on posterior view
Right side	2.520 cm SD=0.851	2.929 cm SD=0.829
Left side	2.569 cm SD = 0.869	2.965 cm SD= 0.904
Male right side	2.551 cm SD=0.8430	2.950 cm SD=0.80243
Male left side	2.619 cm SD= 0.8770	3.008 cm SD= 0.91426
Female right side	2.426 cm SD =0.8713	2.863 cm SD =0 .9049
Female left side	2.416 cm SD = 0.7725	2.834 cm SD= 0.8597

**Figure 3:**
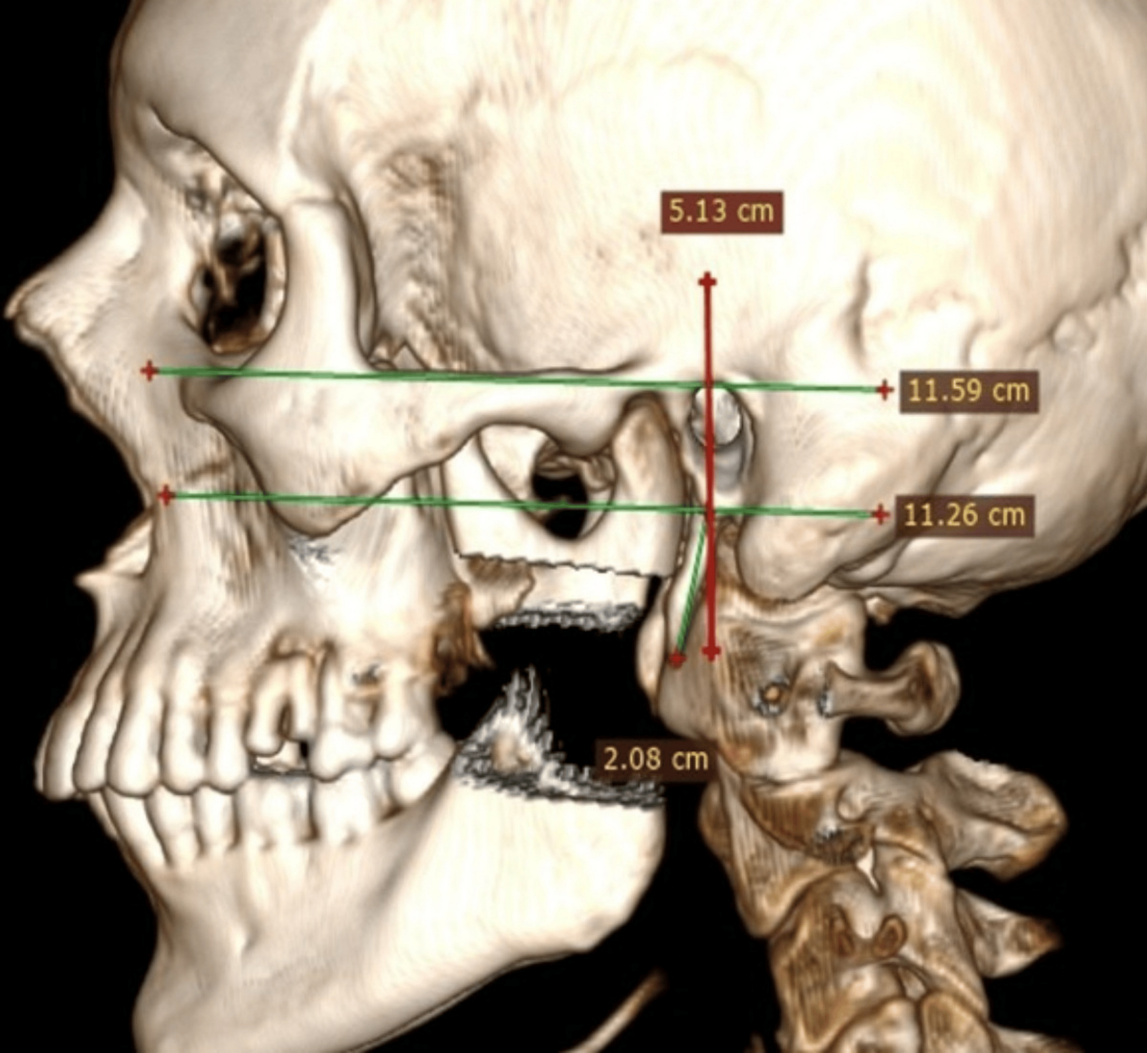
Normal length (<3 cm long) of styloid process on lateral view on 3D CT.

**Figure 4:**
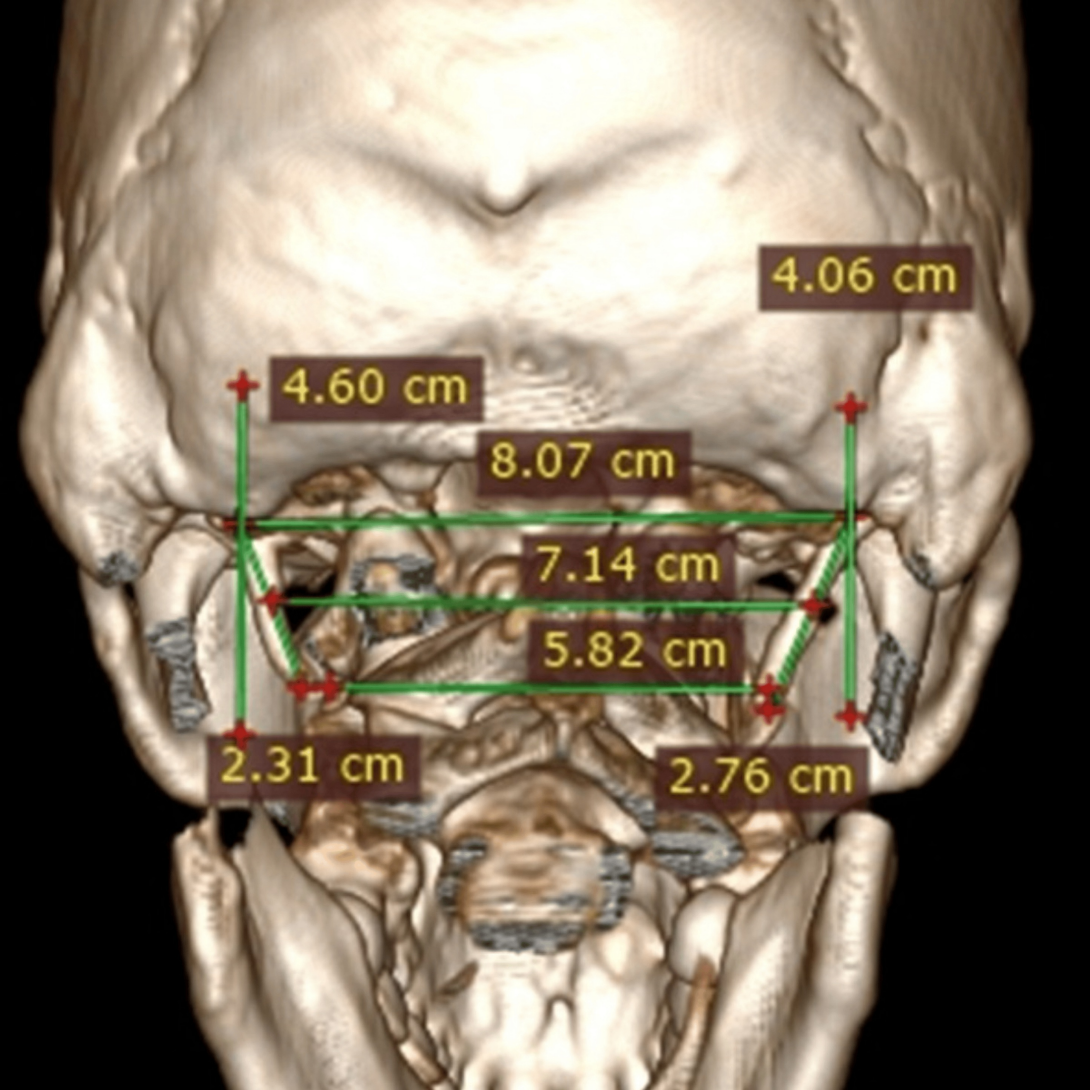
Normal length (<3 cm long) of styloid process on posterior view on 3D CT. 3D CT, three-dimensional computed tomography.

The length of the styloid process was measured using software from the centre of the base to the tip on the lateral (Figure 3) and posterior (Figure 4) 3D CT views.

A styloid process longer than 3 cm was termed an elongated styloid process. It was found in 30% on the lateral view, in contrast, in 53% on the posterior view (Table 4). The difference in the frequency of an elongated styloid process between the lateral and posterior views of 3D CT is statistically significant, with χ²=16.8 and P-value=.000042.

**Table 4:** Shows frequency of elongated styloid process (>3 cm) on lateral and posterior view of 3D CT. 3D CT, three-dimensional computed tomography.

Length of styloid process	Number of cases on the lateral view	Number of cases on the posterior view	Chi-square test	P-value	Degree of freedom
Elongated	45 (30%)	80 (53.3%)	16.8	0.000042	1
Normal	105 (70%)	70 (46.7%)			
Total	150 (100%)	150 (100%)			

In females, the frequency of ESP was 32.4% on the lateral view 48.6% on the posterior view. In males, the frequency of ESP was 29% on the lateral view and 54.9% on the posterior view (Table 5). These findings are not statistically significant, with χ²​​​​​​​=0.2741 and P-value=0.60057 In the age group of 18 to 40, the styloid process was elongated in 28.5% on the lateral view and in 64.2% on the posterior view, 41 - 60 years, ESP was found in 21.6% on lateral view, and in 25% on posterior view and in 61 to 80 years age group, ESP was found in 38.6% on lateral view and in 58% on posterior view (Table 5). These findings are statistically not significant (χ²​​​​​​​=0.8487 and P-value=0.6542).

**Table 5:** Frequency of ESP in both genders and different age groups on posterior and lateral view on 3D CT. ESP, elongated styloid process; 3D CT, three-dimensional computed tomography.

Gender wise ESP	Frequency of ESP on lateral view	Frequency of ESP on posterior view	Total	Chi-square	P-value	Degree of freedom
Female	12 (32.4%)	18 (48.6%)	37 (24.67%)	0.2741	0.60057	1
Male	33 (29.2%)	62 (54.9%)	113 (75.33%)			
Total	45	80	150 (100%)			
Age group wise ESP						
18 to 40	12 (28.5%)	27 (64.2%)	42 (28%)	0.8487	0.65420	2
41 to 60	11 (21.6%)	20 (39.2%)	51(34%)			
61 to 80	22 (38.6%)	33 (57.9%)	57 (38%)			
Total	45	80	150 (100%)			

**Figure 5:**
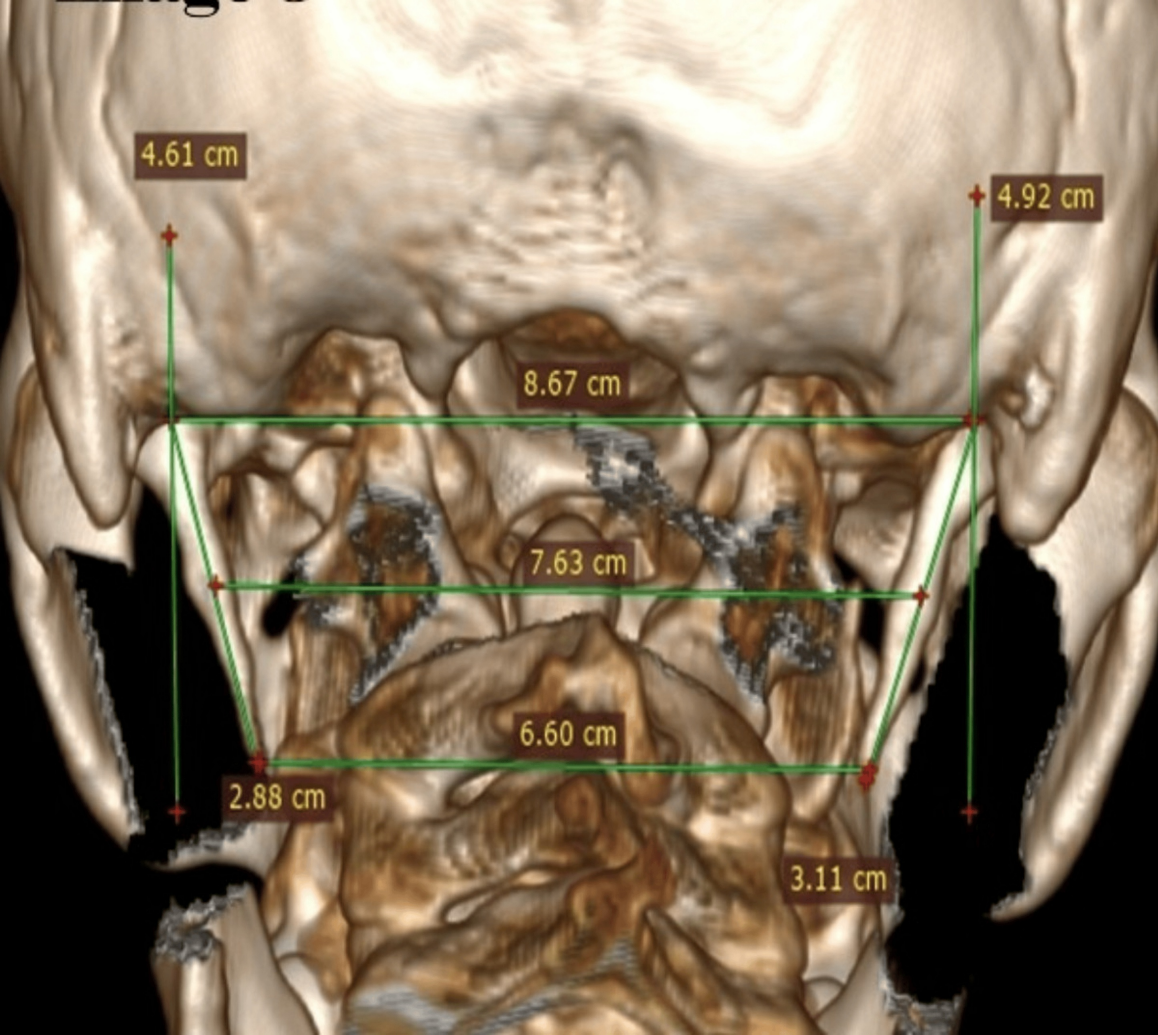
Unilaterally (>3 cm) elongated styloid process on posterior view of 3D CT. 3D CT, three-dimensional computed tomography.

**Figure 6:**
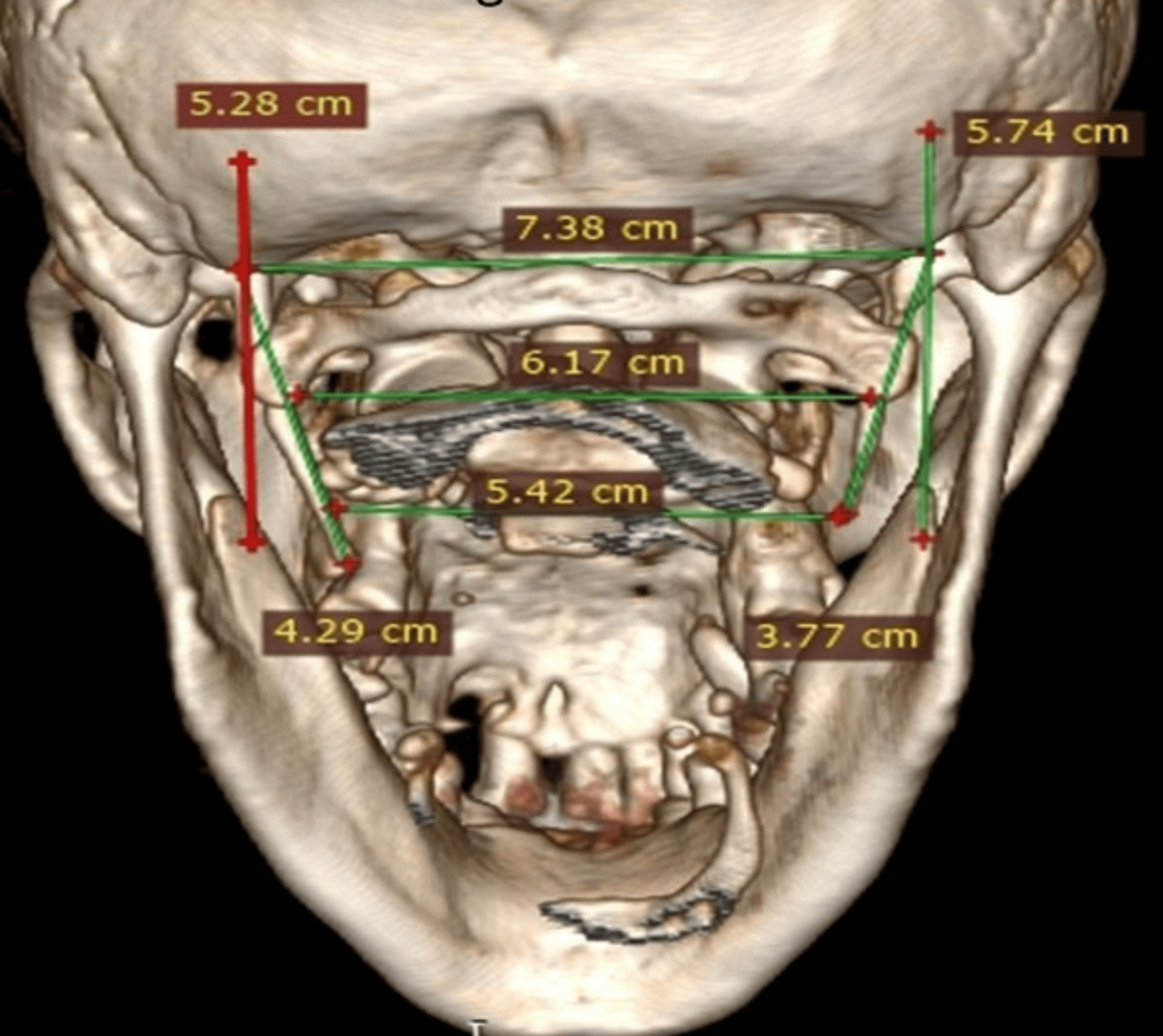
Bilaterally ( >3 cm) elongated styloid process on posterior view of 3D CT. 3D CT, three-dimensional computed tomography.

On the lateral view, the frequency of ESPs was 17.3% bilaterally and 12.7% unilaterally. On the posterior view, frequency was 34% bilaterally and 19.3 unilaterally (Figures 5, 6; Table 6). For laterality, these findings are not statistically significant (Chi-square test χ²​​​​​​​=0.43430. Sidewise frequency of ESP was 22.7% on the left and 21.3% on the right in the lateral view. It was 41.3% on the left side and 45.3% on the right side (Table 6). For the left and right side, χ²​​​​​​​=0.2560 and P-value=0.6128. This difference between the two sides of both views is not statistically significant.

**Table 6:** Shows frequency of ESP on different sides and laterality of ESP on posterior and lateral view. ESP, elongated styloid process.

Sides/laterality	Lateral view		Posterior view	Chi-square test	P-value	Degree of freedom
Left side	34 (22.7%)		62 (41.3%)	0.2560	0.61287	1
Right side	32 (21.3%)		68 (45.3%)			
Unilateral		19 (12.7%)	29 (19.3%)	0.4343	0.50990	1
Bilateral		26 (17.3%)	51 (34%)			
Total		150	150			

## Discussion

The styloid process, a part of the temporal bone, extends downward from its origin to a variable length. The length of the styloid process ranged from less than 1 cm to more than 6 cm, sometimes up to 8 cm long. This length varies among individuals, between the two sides in the same individual, across genders, and across age groups. The frequency of the elongated styloid process also varies by age group, gender, side, and laterality. Many studies have used methods such as CT, CBCT, or 3D CT to ascertain the frequency/prevalence of ESP and the morphometric variations of SP, while others have used digital panoramic radiographs, and others have used a dry skull.

The present study done on 3D CT scan, reported a mean age of 52.32 years (SD = 18.18), Dursun et al. reported a mean age of 46.25 years (SD = 24.58) in their study of 259 3D CT scans [5], Mathur et al. reported a mean age of 43.8 years (SD = 16.7) on 402 MDCT scans (multidetector tomography scans) [11]. Priyadharshini et al. reported a mean age of 40.8 years (SD = 13.9) in 120 CBCT scans (cone-beam tomography scans) [13]. Rokka & Chaudhary reported a mean age of 51 years (SD = 19) across 153 CT scans [15]. Shah et al. reported a mean age of 40.5 years (SD = 7.4) on 304 CT scans [17]. 

Assiri Ahmad H. et al. conducted a study of 400 panoramic radiographs, but the mean age was not calculated [2]. Aoun et al. analysed 489 digital panoramic radiographs and reported a mean age of 47.56 years (SD = 16.15) among Lebanese individuals [6]. Chen et al. reported a mean age of 48.2 years (SD 17.7) in a retrospective analysis of 539 digital panoramic radiographs of Taiwanese people [7]. Hettiarachchi et al. analysed 100 panoramic images but did not report the mean age [8]. The mean age of all the above studies was lower than that of our study. Some authors have conducted their studies on dry skulls to ascertain the length and anatomical variation of the styloid process, but the age range, mean age, age groups, and gender differences could not be determined. Custodio et al. conducted their study on 109 dried skulls for morphological analysis of the styloid process, and Patil et al. conducted their study on 114 dried skulls for morphological study of the styloid process [3,12]. Sheikh & Mittal studied 38 dry skulls, and Vadgaonkar et al. conducted a study on 110 dried skulls for morphological evaluation of the styloid process [16,20]. Vieria et al. reported a mean age of 35.03 years (SD = 17.76) in their study of 736 digital panoramic radiographs [21].

Length range of the styloid process

In our study, the length of the styloid process was found to range from 0.994 to 5.83 cm on the right posterior view,1.01 to 6.3 cm on the left posterior view, 0.495 to 5.65 cm on the right lateral view, and 0.525 to 6.19 cm on the left lateral view. This length range is not significantly different among different views of 3D CT. The similar length range was reported by Custodio et al. who reported a range of length of SP on right posterior view from 1.564 to 6.944 cm on left posterior view, from 1.557 to 6.951cm on left lateral view, 1.022 to 6.973 cm on right lateral view and from 0.830 cm to 6.377 cm on dry skulls, and Rokka & Chaudhary who reported the length range on the right lateral side was 1.8 to 5.54 cm, and on the left lateral side, 1.45 to 5.69 cm on CT scan [3,15]. The following authors reported a narrower length range: Chen et al. reported 1.39-5.00 cm in their study of digital panoramic radiographs [7]; Mathur et al. reported a length range of the styloid process of 0.78 cm to 3.55 cm in males, 1.195 cm to 3.205 cm in females [11]. We have not subclassified the length range separately in both genders.

Mean length of the styloid process

In our study, we found the mean length of the styloid process was 2.929 cm (SD=0.829) on right posterior view, 2.965 cm (SD= 0.904) on left posterior view, 2.520 cm (SD=0.851) on right lateral view and 2.569 cm (SD= 0.856) on left lateral view. Custodio et. al. reported the mean length of the styloid process to be 26.04 mm (SD = 1.265) on the right posterior view, 24.98 mm (SD =1.294) on the left posterior view, 18.90 mm (SD = 1.314) on the right lateral view and 19.25 mm (SD = 1.454) on the left lateral view in 15 dry skulls [3]. Dursun et. al. reported 23.65 mm (SD = 9.51) on the right anterior view, 23.35 mm (SD = .870) on the left anterior view, 21.77 mm (SD =8.82) on the right lateral view, and 21.64 mm (SD =8.11) on the left side on 3D CT [5]. These findings are similar to our findings. Hettiarachchi et al. reported that the mean length of the styloid process was 26.9 mm (SD = 5.4) on the right and 25.5 mm (SD = 5.5) on the left, as measured on digital panoramic radiographs [8]. Mathur et al. reported mean lengths of 23.74 mm (SD=4.50) on the right and 22.74 mm (SD=3.72) on the left on the CT scan [11]. Sheikh & Mittal studied 38 dry skulls and reported the mean length of 1.83 cm (SD = 0.694) on the right posterior view, 1.686 cm (SD = 0.624) on the left posterior view, 1.426 cm (SD = 0.687) on the right lateral view, and 1.155 cm (SD = 0.576) on the left lateral view [16]. Vadgaokar et al. reported that the mean length of SP was 17.8 mm (SD = 9.3) on the right and 18.2 mm (SD = 5.6) on the left, but they did not specify whether these measurements were taken on the lateral or posterior side [20]. These findings are lower than ours; this may be due to the small sample size and the possibility of bone demineralisation. Shah et al. reported a mean length of the styloid process of 31.1 mm (SD = 0.422) on the right side and 31.6 mm (SD = 4.7 cm) on the left side [17]. Shah’s findings of the mean length of the styloid process were higher than our findings. Chen et al. in their study on digital panoramic radiograph reported mean SP length on the right side, 30 mm (SD=0.7) and on the left side, 29.0 mm (SD=0.7) [7]. Priyadarshini et al., in their study on 120 CBCT, reported mean length in males was 3.5 cm (SD= 1.44) on the right side and 3.2 cm (SD=1.25) on the left side, in females 2.7 cm (SD=0.59) on the right side and 2.8 cm (SD= 0.75cm) on the left side [13]. Rokka & Chaudhary reported that the mean length of SP on the right lateral side was 3.06 cm (SD = 0.72), and on the left lateral side, 3.08 cm (SD = 0.72) [15]. 

Frequency of different lengths of the styloid process

To measure the frequency of different lengths of the styloid process, both sides of the styloid process were counted separately in our study. We found that the frequency of styloid processes measuring 2.01-3.00 cm was highest in both the lateral (59%) and posterior (46.3%) views. The second most common length of the styloid process was 3.01 cm to 4 cm, on the lateral view (16.7%) and on the posterior view (34%). The maximum number of styloid processes with normal length was measured 2.01 to 3 cm, and the maximum number of elongated styloid processes was measured 3.01 to 4 cm long. This subclassification of styloid process length has not been reported by other authors.

Frequency of the elongated styloid process

We measured the length of the elongated styloid process on both the lateral and posterior 3D CT views and found an ESP in 30% of 3D CTs on the lateral view and in 53% on the posterior view. In our study, the frequency of an elongated styloid process on the posterior view of 3D CT is statistically significant (p-value = .000042). The frequency of ESP was higher on the posterior view of 3D CT because the size of the tympanic plate of the temporal bone obscures the upper part of the styloid process on the lateral view. The frequency of ESP in our study is close to that reported by Dursun et al., who reported ESP in 43.89% on 3D CT, Priyadarshini et al., who reported 41.7% ESP in their study on 120 CBCT, and Vieira et al., who reported ESP in 43.89 % of OPG [5,13,21]. There are studies that report different prevalence or frequency of ESP. The studies reporting higher prevalence include Assiri Ahmad et al., who reported a 72.75% prevalence of ESP in their study of 400 OPG [2]. Authors who reported a lower prevalence of ESP are Aoun et al., who reported ESP in 15.5% of cases in their study of 489 digital panoramic images, and Hettiarachchi et al., who reported a 29% frequency of ESP in their study of 100 panoramic images [6,8]. Patil et al. reported an ESP prevalence of 14% [12]. Neither of the above authors has mentioned the lateral and posterior length.

Gender-wise frequency of the elongated styloid process

In our study, ESP was found in 32.4% (12) of 3D CT scans on the lateral view and in 48.6% (18) of 3D CTs on the posterior view in females. In males, ESP was found in 29% (33) of 3D CT on the lateral view and in 54.9% (62) of 3D CT on the posterior view. The frequency of ESP is higher in males in our study, but this difference in frequency of ESP between the two genders is not statistically significant in both lateral (chi-square = 0.1384 and p-value = 0.70989) and posterior view (chi-square = 0.4331 and p-value = 0.51048). Studies that reported similar findings of higher prevalence or frequency of ESP include Hettiarachchi et al., who showed a prevalence of ESP of 24.6% in females and 34.9% in males in their study on 100 panoramic images; Priyadarshini et al., who reported a prevalence of ESP of 35.8% in females and 47.4% in males; and Rokka & Chaudhary, who reported that 41.67% of females and 62.85% of males had ESP [8,13,15]. In contrast, a few authors have also reported a higher prevalence of ESP in females. Assiri Ahmad et al. reported an elongated styloid process in 43.64% of males and 56.36% of females; Dursum et al. reported a frequency of ESP of 25.54% in females and 18.35% in males; Aoun et al. reported 15.5% ESP in 489 digital panoramic views, with 59.2% female and 40.8% male; and Vieira et al. documented a frequency of 25.54% ESP in females and 18.35% in males [2,5,6,21].

Age-wise frequency of the elongated styloid process

In our study, the age-wise 3D CTs were divided into three groups, In age group of 18 to 40 years, ESP was found in 28.5% cases on lateral view and in 64.2% of cases on posterior view, in age group of 41 to 60 years ESP was found in 21.6% cases on lateral view, and in 39.2% cases on posterior view and in age group of 61 to 80 years, ESP was found in 38.6% of cases on lateral view and in 58% cases on posterior view. In our study, the frequency of ESP was higher in the 61 to 80-year age group on both the lateral and posterior views. 

Assiri Ahmad H. et al. divided participants into three age groups: 18 to 30, 31 to 60, and above 60 years, reported 66.7% ESP in the 18-30-year age group, 76.6% in the 31-60-year age group, and 72.7% in the> 60-year age group [2]. Aoun et al. divided their study group into 10-year age groups and reported the highest prevalence of ESP (28%) in the 45-64 age group [6]. Vieira et al. reported that among those with ESP, 3.80% were under 17 years, 15.21% were 18 to 35 years, 16.21% were 36 to 53 years, and 8.69% were over 54 years [21]. The age-group formation criteria vary across studies; findings are not comparable with those of others.

Side-wise frequency of elongated styloid process: In our study, we found an elongated styloid process in 22.7% of cases on the left lateral view, 41.3% on the left posterior view, 21.3% on the right lateral view, and 45.3% on the right posterior view. The frequency of elongated styloid process was higher on the right side but not significantly different (Chi-square test - 0.0777 and P value - 0.7804 for lateral view; for posterior, χ² = 0.4887 and P value=.484513). Aoun et al., who reported ESP in 15.5% of 489 digital panoramic views, found that 59.25% had left-sided ESP and 40.8% had right-sided ESP [6]. The percentage reported in this study depends on the total number of ESP, which differs from our study. Chen et al. reported 41.5% ESP on the right and 36.2% on the left in their study of 539 OPGs, with a higher rate on the right [7]. 

Laterality of elongated styloid process: In our study, the styloid process was bilaterally elongated in 17.3% of cases on the lateral view and 34% on the posterior view. It was unilaterally elongated in 12.7% of cases on the lateral view and 19.3% of cases on the posterior view. The difference in unilateral and bilateral frequency of ESP was not significant on the lateral view (χ² = 1.281 and P value = 0.2577) but significant on the posterior view (χ² = 8.25 and P value = .004075). Dursum et al. reported a unilateral elongated styloid process in 7.61% and a bilateral elongated styloid process in 36.28% in their study of 259 3D CT images [5]. The frequency of bilaterally elongated SP of Dursun et al. is similar to our findings in the posterior view, whereas the frequency of unilaterally elongated SP is lower than our findings [5]. Hettiarachchi et al. reported 11% bilateral and 18% unilateral elongation of the styloid process on digital panoramic radiographs [8]. The prevalence of ESP is lower than that reported in our findings. Rokka & Chaudhary reported that in the elongated styloid process, 35.5% of males had unilateral ESP and 64.5% had bilateral ESP, while 30% of females had unilateral ESP and 70% had bilateral ESP on CT scan [15]. Vieira et al. reported that 43.89% had ESP, 36.28% had bilateral ESP, and 7.61% had unilateral ESP on digital panoramic radiographs [21]. The frequency of B/L ESP is similar to our findings, but unilateral ESP is lower.

The cost and availability of a CT machine with 3D conversion software are the main limitations. 3D CT, though a gold standard for measuring the morphometric variation of the styloid process, including length, cannot be used as a screening process.

## Conclusions

It can be concluded that the length of the styloid process varies among different age groups, between males and females, and between the right and left sides of the same individual. The frequency of the elongated styloid process is higher in the posterior view than in the lateral view on 3D CT. This may be due to the tympanic plate, which hides the upper part of the styloid process on the lateral view. The posterior length (length on the posterior view) of the styloid process on 3D CT should be used for the estimation of the frequency of elongated styloid processes. More than 92% of the styloid processes were less than 4 cm in length. It may be the cause of the lesser frequency of occurrence of symptoms of Eagle's syndrome in spite of the higher frequency of elongated styloid processes. The elongation of the styloid more than 3 cm is not the only criterion for the occurrence of symptoms of Eagle’s syndrome. The person with an elongated styloid process of 3.01 cm to 4.00 cm may not develop the symptoms of Eagle's syndrome. The frequency of an elongated styloid process on 3D CT is more accurately measured than with other methods, such as OPG, CBCT, and NCCT. The 3D CT is the gold standard for measuring the length of the styloid process and estimating the frequency of an elongated styloid process.
